# Hydroxychloroquine-Induced Cardiomyopathy: A Case Report

**DOI:** 10.7759/cureus.77763

**Published:** 2025-01-21

**Authors:** Ahmed Hussein, Aya Salih, Tebyan Mohammedali, Yousif Mohamed, L. Maximilian Buja

**Affiliations:** 1 Pathology and Laboratory Medicine, University of Texas Health Science Center, McGovern Medical School, Houston, USA; 2 Internal Medicine, University of Khartoum, Khartoum, SDN; 3 Radiology, University of Khartoum, Khartoum, SDN

**Keywords:** cardiomyopathy, electron microscope, endomyocardial biopsy, fabry disease, heart failure, hydroxychloroquine, mri cardiac

## Abstract

Hydroxychloroquine (HCQ) is an immunomodulatory medication used for decades. HCQ is a weak base accumulating in acidic cytoplasmic vesicles, such as lysosomes. This accumulation raises the pH and interferes with the functions of the lysosomal enzymes, including α-galactosidase A, resulting in a unique clinical and pathologic feature. We present a case with severe HCQ-induced cardiomyopathy to show the pattern of cardiac involvement and available imaging and pathologic modalities. The case involves a 76-year-old woman with a history of Sjögren's disease who had been treated with HCQ for 13 years and presented with symptoms of heart failure. An echocardiogram showed thick ventricles with reduced ejection fraction, and cardiac MRI revealed mid-wall, subepicardial, and patchy late gadolinium enhancement with a non-ischemic distribution, lack of edema and perfusion abnormality, left ventricle wall thickening, biventricular hypokinesis, bilateral atrial enlargement, and mild to moderate mitral valve and tricuspid valve regurgitation, which are compatible with HCQ-induced cardiomyopathy. An endomyocardial biopsy was performed. Light microscopy showed myocyte vacuolization. The periodic acid-Schiff stain was positive. A decrease in color intensity within the vacuoles was observed when diastase was added. Electron microscopy demonstrated inclusion bodies, described as myeloid and curvilinear bodies, confirming the diagnosis. HCQ-induced cardiomyopathy is a rare, potentially fatal side effect. Given the frequent use of this medication, it is important to consider it while evaluating patients with unexplained deterioration in cardiac function after prolonged use.

## Introduction

Hydroxychloroquine (HCQ) is an immunomodulatory medication used for decades to treat various autoimmune and inflammatory conditions [1-3]. This drug functions as a weak base, accumulating in acidic cytoplasmic vesicles, such as lysosomes, and raising the pH of these organelles. This alteration in pH interferes with the normal function of lysosomal enzymes, including α-galactosidase A (α1 Gal A), leading to a unique clinical and pathological presentation [1]. The accumulation of HCQ in lysosomes and its effects on lysosomal enzyme function are well documented in the literature. One of the primary mechanisms by which HCQ impacts lysosomal function is its inhibition of V-ATPase, a key protein responsible for maintaining the acidic pH of lysosomes. This inhibition results in a rise in lysosomal pH, impairing the activity of enzymes like α1 Gal A, which require an acidic environment for optimal function [4].

The clinical implications of HCQ's effects on lysosomes can vary depending on the specific condition being treated. In some instances, the disruption of lysosomal enzyme function may lead to accumulating substrates, which can cause cellular damage and lead to unique clinical features. For example, in patients with Fabry disease, an inherited lysosomal storage disorder, HCQ has been shown to exacerbate symptoms by further impairing the activity of α1 Gal A [4]. In addition to its effects on lysosomal enzymes, HCQ has been associated with cardiac toxicity, manifesting as arrhythmias, conduction disturbances, and cardiomyopathy [1-2]. The underlying pathophysiology of HCQ-induced cardiomyopathy is not fully understood but is thought to involve direct myocardial toxicity from the drug and its metabolites accumulating within the myocardium. Although genetic predisposition has not been well established, the dose and duration of HCQ exposure may influence this [3,5].

While retinopathy is a well-known side effect of this medication, cardiac complications are less frequently reported. The imaging findings associated with HCQ-induced cardiomyopathy can be distinctive, though not pathognomonic, requiring an interdisciplinary approach that includes clinical correlation and, if necessary, biopsy for definitive diagnosis [5]. Characteristic imaging findings in HCQ-induced cardiomyopathy include mid-wall, subepicardial, and patchy late gadolinium enhancement on cardiac magnetic resonance imaging, usually with a non-ischemic distribution [3]. These findings indicate myocardial fibrosis or replacement, resulting in biventricular hypokinesis, bilateral atrial enlargement, and mild to moderate mitral and tricuspid regurgitation. Notably, these imaging findings may lack associated myocardial edema or perfusion abnormalities, helping to distinguish this condition from other forms of myocarditis or cardiomyopathy.

HCQ is commonly prescribed for autoimmune diseases like Sjögren's syndrome, lupus, and rheumatoid arthritis due to its immunomodulatory and anti-inflammatory effects. While generally considered safe, long-term HCQ use carries a risk of cardiomyopathy, a condition where the heart muscle becomes weakened and enlarged, leading to impaired function. This adverse effect can manifest as heart failure with symptoms like shortness of breath, fatigue, and edema.

HCQ-induced cardiomyopathy has an estimated incidence ranging from 0.5% to 2%, although subclinical myocardial changes may be more common [6]. The risk appears to increase with prolonged use (over five years) and higher cumulative doses of HCQ [7]. Other potential risk factors include pre-existing heart conditions, renal insufficiency, hypokalemia, and concomitant use of certain medications like amiodarone or other QT-prolonging drugs [8]. Female sex and older age have also been suggested as potential risk factors. However, it's important to note that HCQ cardiotoxicity can occur even in patients without these risk factors, highlighting the need for vigilance in all individuals receiving long-term HCQ therapy. Genetic factors may also play a role, although research in this area is ongoing.

Clinicians must maintain a high index of suspicion for HCQ-induced cardiomyopathy in patients presenting with unexplained cardiac dysfunction, particularly in those with a history of chronic HCQ use. An interdisciplinary approach incorporating clinical history, laboratory findings, imaging, and potentially endomyocardial biopsy is crucial for accurate diagnosis and effective management [3-5]. Management strategies should focus on early recognition and diagnosis. Healthcare providers should be vigilant for developing cardiac symptoms such as shortness of breath, fatigue, and arrhythmias. If cardiomyopathy is suspected in patients receiving long-term HCQ therapy, the medication should be discontinued immediately to prevent further myocardial damage. Patients may require treatment for heart failure, including diuretics, vasodilators, and antiarrhythmic medications, to manage symptoms and maintain cardiac function. Regular echocardiographic and electrocardiographic monitoring is recommended for patients with suspected or confirmed HCQ-induced cardiomyopathy to assess the progression or regression of the condition.

Overall, prognosis varies and largely depends on prompt diagnosis and management. In this context, we describe a patient who experienced severe cardiomyopathy caused by HCQ, emphasizing the importance of prompt identification and comprehensive diagnostic methods to guide treatment. Our case illustrates the utility of cardiac MRI and endomyocardial biopsy in diagnosing HCQ-induced cardiomyopathy to ensure accurate diagnosis and appropriate management.

This case contributes to the growing body of literature documenting HCQ-induced cardiomyopathy. While other forms of cardiomyopathy, such as dilated, hypertrophic, or restrictive cardiomyopathy, are more prevalent and often associated with distinct etiologies like genetic mutations, coronary artery disease, or infections, HCQ-induced cardiomyopathy represents a unique entity. Its significance lies in the potential for reversibility upon discontinuing the medication, highlighting the importance of early recognition and intervention. This case reinforces the need for vigilance among clinicians prescribing long-term HCQ therapy and emphasizes the value of periodic cardiac monitoring to detect early signs of cardiotoxicity. Further research exploring the underlying mechanisms of HCQ-induced cardiomyopathy and identifying specific risk factors could inform more targeted screening strategies and improve patient outcomes.

## Case presentation

A 76-year-old female patient with a history of Sjogren's disease, who has been receiving HCQ treatment for the past 13 years, presented with bilateral lower extremity swelling, dyspnea on exertion, and chest discomfort. Her medical history is significant for a myocardial infarction (MI) that occurred 10 years prior, without notable coronary artery disease, as well as moderate mitral regurgitation and severe tricuspid regurgitation. In addition to Sjogren's syndrome, the patient also has hypothyroidism and hypertension. Following her MI, the patient initially exhibited a brief period of clinical improvement; however, there was a gradual decline in her condition over several months. She reported experiencing dyspnea with minimal exertion, which subsequently progressed to dyspnea during conversation. Furthermore, the patient encountered frequent episodes of coughing, bilateral lower extremity swelling, and intermittent substernal chest pain. Relevant laboratory findings are presented in the accompanying Table 1.

**Table 1:** Relevant laboratory results pg/mL: picograms per milliliter, ng/ml: nanograms per milliliter, U/L: units per liter, mU/L: milliunits per liter, g/dL: gram per deciliter, U/mL: units per milliliter, BNP: B-type natriuretic peptide, AST: aspartate aminotransferase, ALT: alanine aminotransferase, TSH: thyroid-stimulating hormone, ANA: antinuclear antibodies, Anti-dsDNA: anti-double-stranded deoxyribonucleic acid

Laboratory parameter	Result	Reference interval
BNP	4,617 pg/mL	<100 pg/mL
Troponin I	1,523 ng/mL	0-0.04 ng/mL
AST and ALT	120 U/L	10-35 U/L
ALT	129 U/L	0-30 U/L
TSH	3.4 mU/L	0.4-4.5 mU/L
Total protein	6.5 g/dL	6.0-8.3 g/dL
Albumin	3.2 g/dL	3.4-5.4 g/dL
ANA	1:80, nuclear, speckled	<1:40
Anti-dsDNA antibodies	Negative	<5 U/mL considered negative
Anti-Smith	Negative	<7 U/mL considered negative

A transthoracic echocardiogram demonstrated a left ventricular ejection fraction ranging from 35% to 39%, accompanied by global hypokinesis and grade II diastolic dysfunction. The right ventricle exhibited a normal size but presented with reduced systolic function. Mild mitral regurgitation and tricuspid regurgitation were also noted. During a right heart catheterization, left- and right-sided filling pressures were reported as normal; nevertheless, the patient's cardiac output and index were low. In light of these clinical symptoms and echocardiographic findings, the patient has been diagnosed with heart failure with reduced ejection fraction of uncertain etiology. She was given intravenous diuretics and discharged with an oral diuretic, experiencing a brief improvement before her symptoms returned. Despite increasing her diuretic dose at home, she remained symptomatic. She can only walk about 20 steps before stopping due to shortness of breath. Additionally, she has noticed weight gain, swelling in both lower extremities, and episodes of non-exertional, non-radiating chest pressure, which have occurred three to four times since her discharge, lasting a few minutes at a time and somewhat relieved by sublingual nitroglycerin.

**Figure 1:**
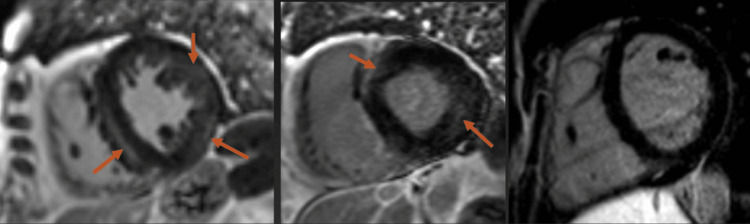
Cardiac MRI with LGE images showing mid-wall, patchy, and epicardial late gadolinium enhancement involving the septum and anterior and lateral walls with subendocardial sparing (arrows), with LGE burden approximately 15% of the left ventricle MRI: magnetic resonance imaging, LGE: late gadolinium enhancement

**Figure 2:**
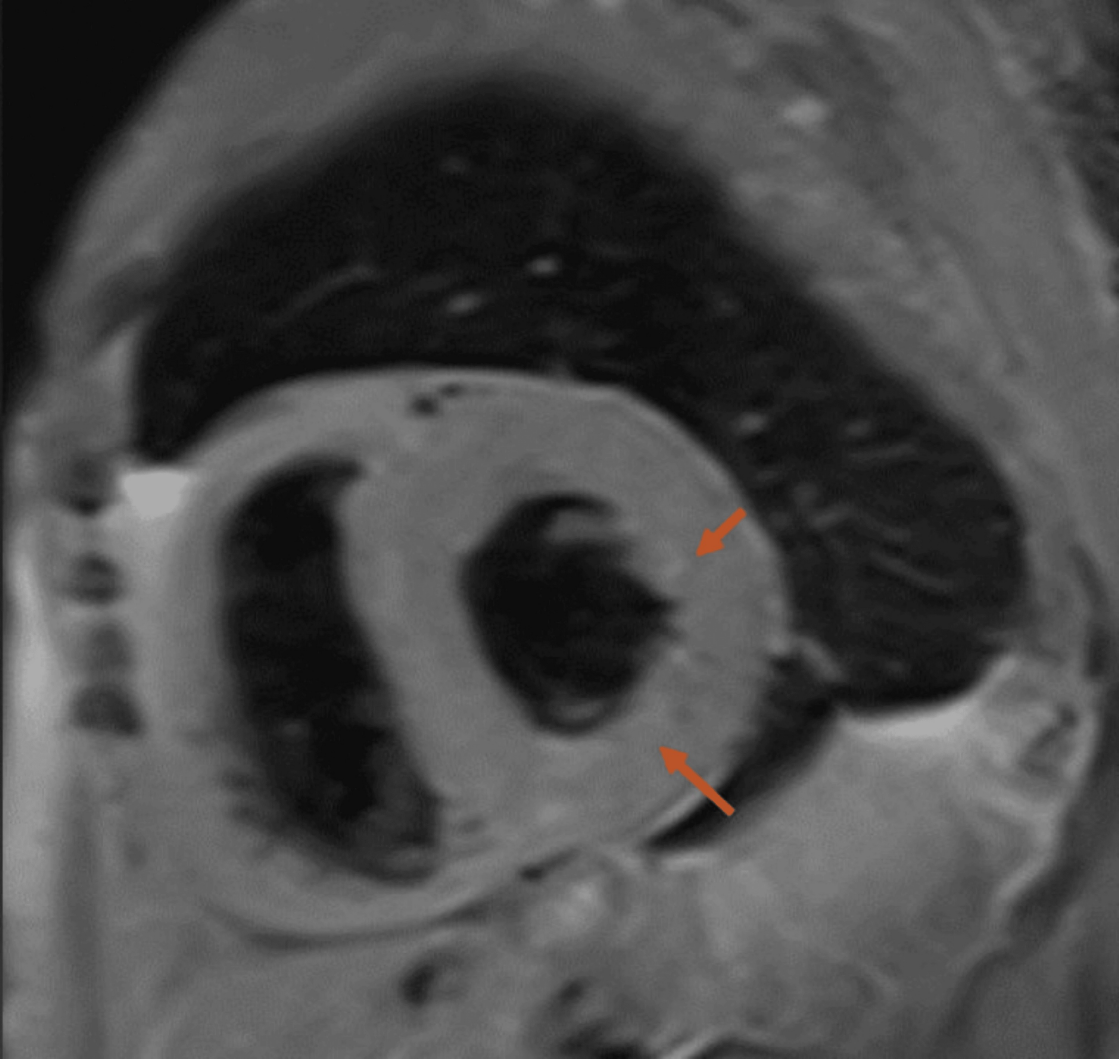
T2-weighted cardiac MRI image revealing concentric hypertrophy (arrows) without myocardial edema. The absence of myocardial edema on cardiac MRI, despite the presence of late gadolinium enhancement and left ventricular wall thickening, is a crucial finding that points toward HCQ-induced cardiomyopathy MRI: magnetic resonance imaging, HCQ: hydroxychloroquine

**Figure 3:**
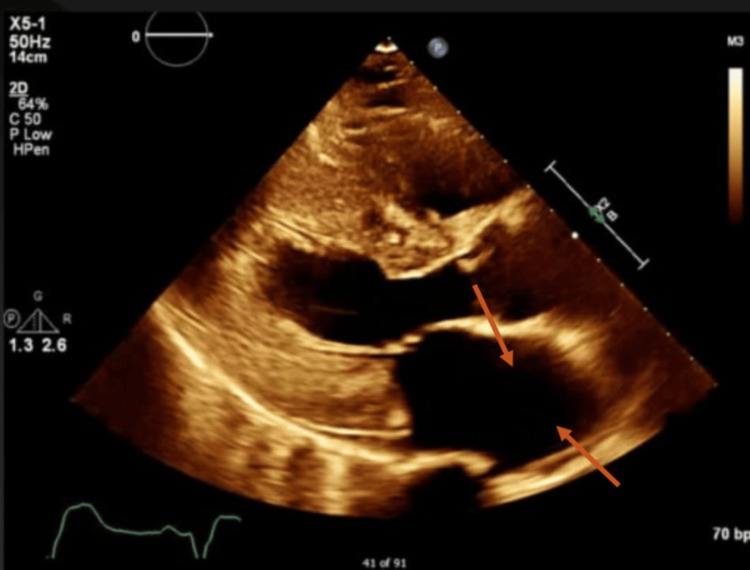
Transthoracic echocardiogram, parasternal long-axis view, showing left atrial enlargement (arrows) and mild diffuse hypokinesis

**Figure 4:**
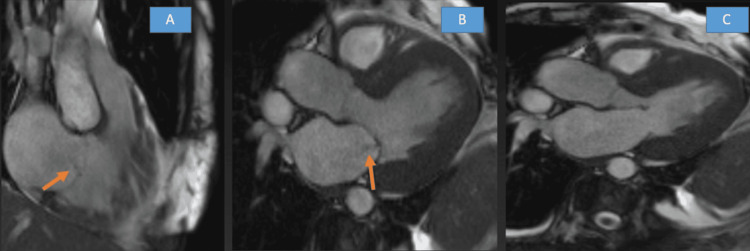
Cardiac MRI with late gadolinium enhancement images showing (A) mild to moderate tricuspid regurgitation (regurgitant fraction 33%), (B) mild to moderate mitral regurgitation (regurgitant fraction 35%), and (C) normal cardiac MRI for comparison MRI: magnetic resonance imaging

**Figure 5:**
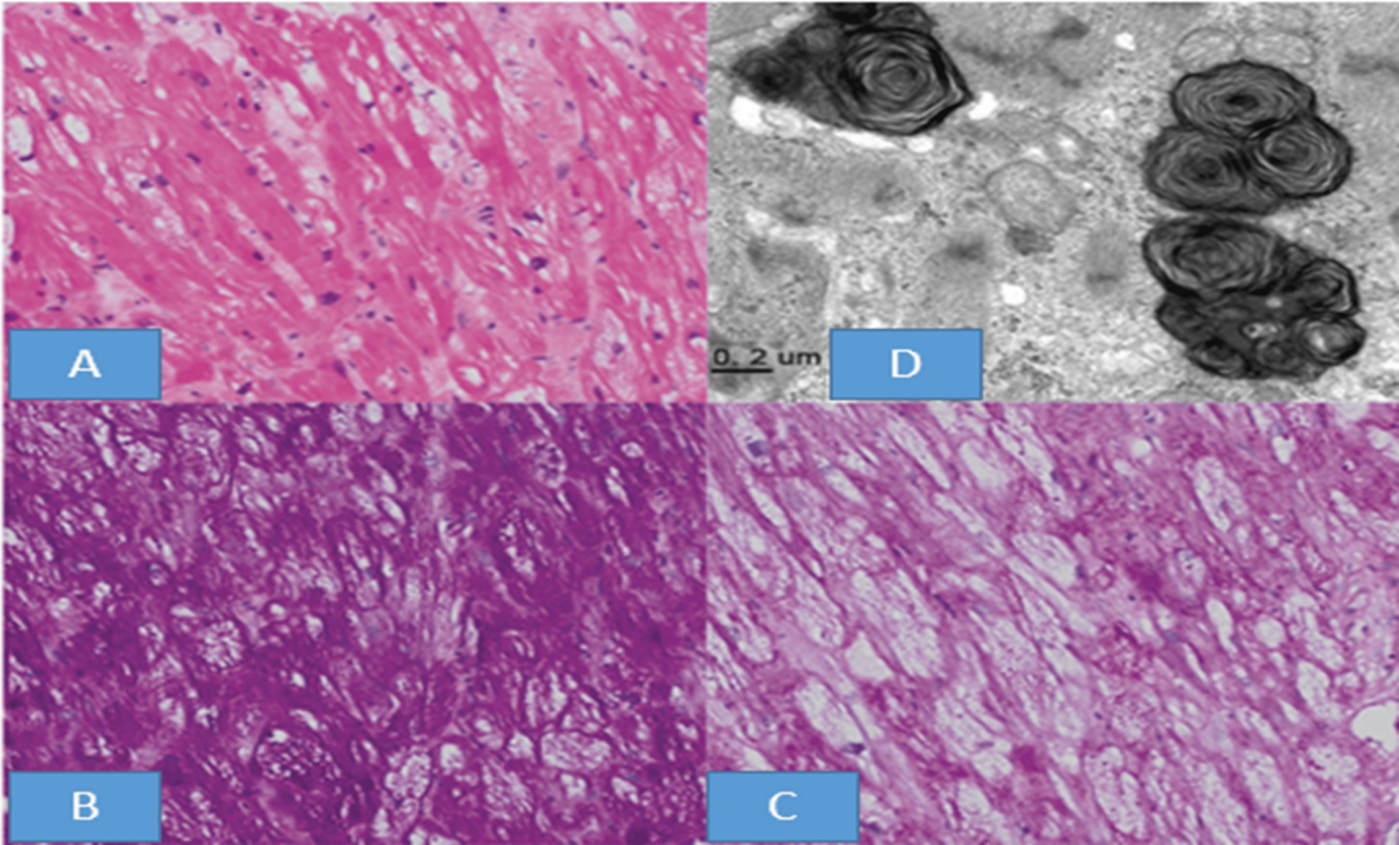
(A) Light microscopy H&E stain (x200) showing myocyte vacuolization, (B) positive periodic acid-Schiff stain (x200), (C) adding diastase to periodic acid-Schiff stain (x200), the color intensity within the vacuoles decreased, and (D) electron microscopy demonstrating inclusion bodies described as myeloid and curvilinear bodies (400x) H&E: hematoxylin and eosin

Additional cardiac imaging, primarily utilizing MRI, revealed mid-wall, subepicardial, and patchy late gadolinium enhancement (Figure 1), characterized by a non-ischemic distribution without perfusion abnormalities (Figure 2) and left ventricular wall thickening (Figure 3). The findings indicate biventricular hypokinesis, bilateral atrial enlargement (Figure 4), mild to moderate mitral valve, and tricuspid valve regurgitation (Figure 5), consistent with HCQ-induced cardiomyopathy. A consultation with the rheumatology team was conducted, leading to the cessation of HCQ treatment. However, it is noteworthy that no pathognomonic imaging features definitive for HCQ-induced cardiomyopathy were identified; thus, consideration was given to performing an endomyocardial biopsy.

The endomyocardial biopsy analysis showed myocyte vacuolization, as observed under light microscopy (Figure 5A). The periodic acid-Schiff stain yielded a positive result (Figure 5B). Upon the addition of diastase, a notable decrease in color intensity within the vacuoles was recorded (Figure 5C). Furthermore, electron microscopy identified the presence of inclusion bodies, characterized as myeloid and curvilinear bodies (Figure 5D). The clinical findings are summarized in Table 2 to make it clear.

**Table 2:** Summary of clinical findings HCQ: hydroxychloroquine, MI: myocardial infarction, CAD: coronary artery disease, BNP: B-type natriuretic peptide, AST: aspartate aminotransferase, ALT: alanine aminotransferase, LVEF: left ventricular ejection fraction, RHC: right heart catheterization, LGE: late gadolinium enhancement, LV: left ventricle, ECHO: echocardiogram

Feature	Findings	Significance
Patient	76F, Sjogren's (13 yrs on HCQ), prior MI (10 yrs ago, no CAD)	Long-term HCQ use raises suspicion for drug-induced cardiomyopathy
Symptoms	Dyspnea, chest discomfort, cough, lower extremity edema, weight gain	Suggestive of heart failure
Key labs	Elevated BNP, troponin, AST/ALT; low protein/albumin	Cardiac stress/injury, possible hepatic congestion
ECHO	LVEF 35-39%, global hypokinesis, grade II diastolic dysfunction	Systolic dysfunction with diffuse myocardial involvement
RHC	Normal filling pressures, low cardiac output/index	Impaired contractility
Cardiac MRI	Non-ischemic LGE, no edema, LV wall thickening	Key diagnostic clue: LGE pattern without edema strongly suggests HCQ cardiomyopathy

Based on the findings from the cardiac MRI and cardiac biopsy, along with the relevant clinical context, a diagnosis of HCQ-induced cardiomyopathy was established. The patient demonstrated improvement with continued diuresis, maintained stable oxygen levels on room air throughout the hospitalization, and was discharged home after an eight-day stay. The outpatient follow-up with the cardiology and pulmonology departments indicated that diuretics were being optimized. Additionally, pulmonary function tests revealed obstructive disease, and a rheumatology follow-up was pending, as was interval imaging. Unfortunately, the patient experienced a collapse at home and passed away despite the efforts of emergency medical services to perform cardiopulmonary resuscitation.

## Discussion

The use of HCQ and chloroquine as long-term maintenance therapies can lead to severe cardiac toxicities, including cardiomyopathy and drug-induced heart failure. Individuals diagnosed with cardiomyopathy due to these medications have ranged in age from 31 to 81 years, with women being more commonly affected. The average duration of treatment was 13 years, with a range of two to 35 years, and large cumulative doses (1277-1843 g) were associated with the development of cardiomyopathy. However, cases of heart failure have also been reported following the use of low-dose chloroquine. In rare cases, the cardiac complications have been severe enough to necessitate heart transplantation.

While HCQ-induced cardiomyopathy's precise prevalence in autoimmune patients remains challenging to determine definitively, estimates vary widely, ranging from less than 1% to as high as 7% in some studies, likely reflecting differences in patient populations, duration of HCQ use and diagnostic criteria [9]. This variability underscores the need for more robust epidemiological studies. Diagnosis often relies on clinical findings (e.g., shortness of breath, edema), electrocardiographic abnormalities, and imaging studies like echocardiography or cardiac MRI. Endomyocardial biopsy, considered the gold standard for confirming the diagnosis, is invasive and not routinely performed [8]. This reliance on less definitive diagnostic methods may lead to underdiagnosis and overdiagnosis, further complicating prevalence estimates. The potential for subclinical myocardial changes also adds to the complexity. These limitations in diagnostic methods can impact the generalizability of findings from existing studies, making it challenging to extrapolate prevalence rates to broader patient populations.

In the context of HCQ-induced cardiomyopathy, abnormal laboratory values can provide further evidence of cardiac dysfunction. Brain natriuretic peptide is a hormone released by the heart in response to stretching or pressure overload, and elevated B-type natriuretic peptide (BNP) levels can indicate heart failure. Similarly, troponin is a protein released into the bloodstream when heart muscle is damaged, and elevated troponin levels, particularly high-sensitivity troponin I, can suggest myocardial injury. While not specific to HCQ cardiotoxicity, these markers, when elevated in conjunction with clinical findings and imaging abnormalities, can support the diagnosis and help assess the severity of cardiac involvement. It's important to note that other conditions can also cause elevations in BNP and troponin, so these markers should be interpreted in the context of the patient's overall clinical picture.

HCQ imaging has emerged as a valuable tool in comprehensively evaluating left ventricular and right ventricular function and morphology. It is crucial in excluding and differentiating various cardiomyopathies, such as myocarditis, ischemic cardiomyopathy, and infiltrative cardiomyopathies [10]. Late gadolinium enhancement on HCQ imaging in non-ischemic cardiomyopathies is a significant predictor of adverse cardiac outcomes, with an eightfold increased risk of adverse events [10]. Additionally, HCQ imaging can serve as a monitoring tool, suggesting the need for earlier referral for device therapy, such as implantable cardioverter-defibrillators or even cardiac transplantation. Cine imaging, a component of HCQ imaging, allows for assessing left ventricular systolic function, which can be altered with changes in ventricular volumes. Phase-contrast sequences in HCQ imaging can also evaluate diastolic function, detecting decreased left ventricular compliance through a narrow blood inflow jet in early diastole and an early peak-atrial peak ratio greater than two [10].

Furthermore, myocardial scarring and signs of micro-ischemia, which are characteristic of hypertrophic cardiomyopathy, can be visualized on late gadolinium enhancement imaging, and the extent of these findings is associated with disease progression and the risk of sudden cardiac death [10]. As a non-invasive and widely available imaging modality, Echocardiography is crucial in the screening and initial diagnosis of pulmonary hypertension. Specific echocardiographic features, such as a dilated main pulmonary artery, enlargement of right-sided cardiac chambers, right ventricular hypertrophy, paradoxical septal motion, and tricuspid regurgitation, can provide valuable information about the presence and severity of pulmonary hypertension.

Additionally, assessing ventricular function through echocardiographic techniques can offer prognostic information and a non-invasive means of monitoring disease progression or response to therapy. Imaging findings such as mid-wall, supepicardial, and patchy late gadolinium enhancement with a non-ischaemic distribution, lack of edema and perfusion abnormality, left ventricular wall thickening, biventricular hypokinesis, biatrial enlargement, mild to moderate mitral valve and tricuspid valve regurgitation are compatible with HCQ-induced cardiomyopathy. Overall, HCQ imaging has become an indispensable tool in the comprehensive evaluation of left ventricular and right ventricular function and morphology, with its ability to provide valuable diagnostic, prognostic, and therapeutic guidance in various cardiomyopathies.

The cardiac biopsy findings in a case of HCQ-induced cardiomyopathy include extensive myocyte vacuolization, a hallmark feature of this condition. The periodic acid-Schiff stain was positive, indicating the presence of glycogen or glycoprotein accumulation within the vacuoles. Interestingly, upon the addition of diastase, the color intensity within the vacuoles decreased, suggesting that the positive staining was primarily due to the presence of glycogen [5]. Electron microscopy of the biopsy specimen further revealed the presence of characteristic inclusion bodies, described as myeloid and curvilinear bodies. These ultrastructural findings are consistent with the accumulation of phospholipids, a common pathological feature in HCQ-induced cardiomyopathy [5,11]. Our case's cardiac biopsy findings align with previously reported cases, which have demonstrated similar histological and ultrastructural changes in the myocardium [3-5,11]. These changes appear to result from the drug's interference with normal lysosomal function, leading to the accumulation of substances within the myocytes and ultimately resulting in myocyte damage and cardiomyopathy. Identifying these characteristic biopsy findings is crucial for diagnosing and managing HCQ-induced cardiomyopathy, as the clinical presentation can be nonspecific and may mimic other forms of cardiomyopathy.

Furthermore, notable ultrastructural similarities exist between the inclusions associated with HCQ toxicity and those observed in Fabry disease. Fabry disease is an X-linked lysosomal disorder characterized by a significant deficiency or complete absence of the enzyme α1 Gal A, leading to the accumulation of glycosphingolipids in various tissues [8]. The inclusions found in Fabry disease are typically described as "myeloid" and "curvilinear" in nature, with a distinct ultrastructural appearance [8]. Interestingly, these same types of inclusions have been reported in cases of HCQ toxicity, suggesting that they may be more specific to this condition than the inclusions seen in Fabry disease [12]. However, a closer examination of the available evidence suggests that the ultrastructural similarities between the inclusions in these two conditions are, in fact, quite striking. Fabry disease and HCQ toxicity exhibit inclusions characterized by a curvilinear, membranous appearance with a distinct lamellar or whorled pattern. The inclusions in HCQ cases are typically periodic acid-Schiff-negative, in contrast to the periodic acid-Schiff-positive inclusions observed in Fabry disease [13]. Other differences are also mentioned when myeloid and curvilinear bodies within cardiomyocytes and interstitial cells are observed using electron microscopy [14]. This lysosomal buildup eventually interferes with the normal function of cells in various organs, including the heart, kidneys, and nervous system [14]. Cardiac manifestations of Fabry disease can include unexplained left ventricular hypertrophy, valvular regurgitation, and conduction abnormalities. These clinical findings can guide the decision to perform a biopsy [15].

The existing literature suggests that in cases where HCQ has been discontinued due to suspected cardiomyopathy, some patients have experienced improvements within three months to five years. However, complete recovery is not guaranteed, and the cardiac side effects of these medications can be challenging to identify due to their often nonspecific clinical presentation. Therefore, the systematic monitoring and identification of potentially affected patients are paramount.

## Conclusions

HCQ is a pharmaceutical agent frequently employed to manage various autoimmune and inflammatory disorders. It is imperative to acknowledge that this medication may induce significant cardiac side effects, some of which can be severe and potentially irreversible, requiring careful consideration in patients on long-term therapy. Early detection and intervention are critical to mitigate the risk of irreversible cardiac damage. Before initiating HCQ therapy, a baseline cardiac evaluation should be performed, including an electrocardiogram and echocardiogram. For patients on long-term HCQ, periodic monitoring with an annual electrocardiogram and echocardiogram is reasonable, especially for those with pre-existing cardiac conditions or other risk factors. Patient education is also essential, emphasizing the potential cardiac risks and the importance of reporting new or worsening symptoms like shortness of breath, fatigue, or edema.

If there is any indication of cardiac damage, promptly discontinue HCQ and pursue additional diagnostic assessments, such as cardiac MRI and endomyocardial biopsy, which can yield vital information. These measures are critical in determining appropriate management strategies and may help facilitate patient recovery. Further research is needed to identify specific risk factors that predispose individuals to HCQ-induced cardiomyopathy, investigate potential biomarkers for early detection, and determine optimal treatment strategies beyond discontinuation of HCQ. Raising awareness and promoting proactive monitoring can minimize the risk of this rare but significant adverse effect and improve the safety of long-term HCQ therapy.
